# Development of a duplex droplet digital PCR assay for the detection of *Burkholderia cepacia* complex and *Stenotrophomonas maltophilia* in bloodstream infections

**DOI:** 10.1128/spectrum.03569-23

**Published:** 2024-02-27

**Authors:** Chunmei Liu, Ziqiang He, Mimi Kong, Dong Jin

**Affiliations:** 1 Department of Epidemiology, School of Public Health, Shanxi Medical University, Taiyuan, Shanxi, China; 2 National Key Laboratory of Intelligent Tracking and Forecasting for Infectious Diseases, National Institute for Communicable Disease Control and Prevention, Chinese Center for Disease Control and Prevention, Beijing, China; 3 Hebei Key Laboratory of Intractable Pathogens, Shijiazhuang Center for Disease Control and Prevention, Shijiazhuang, Hebei, China; University of Pretoria, Pretoria, South Africa

**Keywords:** droplet digital PCR, *Burkholderia cepacia *complex, *Stenotrophomonas maltophilia*, bloodstream infection

## Abstract

*Burkholderia cepacia* complex (BCC) and *Stenotrophomonas maltophilia* are nosocomial pathogens that cause various infections and exhibit high resistance to multiple antimicrobial agents. In this study, we aimed to develop a duplex droplet digital PCR (ddPCR) assay for detecting BCC and *S. maltophilia* in bloodstream infections. We optimized the experimental conditions by setting the annealing temperature to 51°C and determining the optimal concentrations of primers and probes, as well as the thermal cycle numbers. The feasibility of the duplex ddPCR reaction system with the optimal conditions was established and verified through parallel reactions with reference strains of BCC and *S. maltophilia*. The specificity of the assay, tested with 33 reference strains, was found to be 100%. The duplex ddPCR assay demonstrated good repeatability and could detect as low as 5.35 copies/reaction of BCC and 7.67 copies/reaction of *S. maltophilia*. This level of sensitivity was consistent in the simulated blood and blood bottle samples. We compared nucleic acid extraction methods and found that the Chelex-100 boiling method and kit extraction method exhibited similar detection sensitivity, suggesting the potential application of the Chelex-100 boiling method in the ddPCR assay. In the clinical samples, the duplex ddPCR assay accurately detected BCC and *S. maltophilia* in 58 cases. In conclusion, our study successfully developed a duplex ddPCR assay that provides accurate and convenient detection of BCC and *S. maltophilia* in bloodstream infections.

**IMPORTANCE:**

*Burkholderia cepacia* complex (BCC) and *Stenotrophomonas maltophilia* are implicated in a wide range of infections, including bloodstream infections (BSIs), pneumonia, and meningitis, and often exhibit high intrinsic resistance to multiple antimicrobial agents, limiting therapeutic options. The gold standard for diagnosing bloodstream infections remains blood culture. However, current blood culture detection and positivity rates do not meet the “rapid diagnosis” required for the diagnosis and treatment of critically ill patients with BSIs. The digital droplet PCR (ddPCR) method is a potentially more powerful tool in the diagnosis of BSIs compared to other molecular methods due to its greater sensitivity, specificity, accuracy, and reproducibility. In this study, a duplex ddPCR assay for the detection of BCC and *S. maltophilia* in BSIs was developed.

## INTRODUCTION

Nonfermenting Gram-negative bacilli (NFGNB), such as *Pseudomonas aeruginosa*, *Acinetobacter baumannii*, *Burkholderia cepacia* complex (BCC), and *Stenotrophomonas maltophilia*, are an important group of nosocomial pathogens that contribute to significant morbidity and mortality. They are implicated in a wide range of infections, including bloodstream infections (BSIs), pneumonia, and meningitis, and often exhibit high intrinsic resistance to multiple antimicrobial agents, limiting therapeutic options (1, 2). While *P. aeruginosa* and *A. baumannii* continue to be the most prevalent species among NFGNB in clinical settings (1, 3, 4), it is important to note the emergence of other species, particularly BCC and *S. maltophilia*, as significant nosocomial pathogens. BCC and *S. maltophilia* are frequently isolated from patients in intensive care units as well as those with cystic fibrosis (4). Additionally, they have been implicated in hospital-acquired bloodstream outbreaks (5, 6).

BSIs, as a severe systemic infectious disease, are prone to induce sepsis and multiple organ dysfunction syndrome, with a high mortality rate, and have become one of the major global public health burdens (7). The appropriate management of BSI patients is closely related to the timely and accurate detection of the pathogens, as well as the rational use of initial antimicrobial drugs (8). The gold standard for diagnosing bloodstream infections remains blood culture. However, current blood culture detection and positivity rates do not meet the “rapid diagnosis” required for the diagnosis and treatment of critically ill patients with BSIs. Approximately 35% of BSIs or sepsis cases are challenging to identify the pathogen through conventional culture methods, leading to the occurrence of culture-negative sepsis. This increases the misdiagnosis rates of BSIs, ultimately resulting in a poor prognosis for critically ill patients (9). The administration of broad-spectrum antimicrobial therapy prior to obtaining blood culture specimens has been shown to increase the incidence of false-negative results in blood cultures (10). Moreover, the use of such antibiotics may also contribute to false-negative results in certain infections caused by BCC and *S. maltophilia* (11–13). These issues, along with bacterial contamination, pose challenges in terms of accurate detection and culture methods for both false-positive and false-negative results (14). With the development and clinical application of molecular technologies, such as matrix-assisted laser desorption time of flight mass spectrometry, metagenomics second-generation sequencing (mNGS), and droplet digital PCR (ddPCR) in clinical microbiology laboratories, the process of detecting bloodstream infections from sampling to reporting has been further optimized (15–17). The mNGS method has been developed to detect pathogens in blood by analyzing fragments of microbial cell-free DNA present in plasma (18, 19). When compared to the traditional culture method, the mNGS technique demonstrated a significantly higher sensitivity rate of 52.7% as opposed to 34.4% (19). Interestingly, the mNGS method was also found to be less affected by prior antibiotic usage when compared to the traditional culture method (19).

The ddPCR method is a potentially more powerful tool in the diagnosis of BSIs compared to other molecular methods due to its greater sensitivity, specificity, accuracy, and reproducibility (17, 20). ddPCR is performed by dispensing a mixture of reagents containing a DNA-binding fluorescent dye into thousands of individual reaction droplets in an oil emulsion and then performing amplification. The number of target nucleic acids is determined by counting the positive or negative droplets of the target amplification based on the fluorescence of the DNA-binding dye. The distribution of positive droplets is processed using Poisson statistics to assess the concentration of target nucleic acids in each sample without the use of a standard curve (21–23). As a result, one of the main benefits of ddPCR compared to other molecular methods is its ability to perform absolute quantification of target genes without the need for standards, references, or calibration curves. This feature is particularly attractive for the rapid detection of BCC and *S. maltophilia* infections (24, 25). DNA extraction from blood samples has always been challenging due to the low infection dose and the presence of inhibitors for PCR amplification (26). The ddPCR method, which offers the advantage of high sensitivity and tolerance to inhibitors also, provides an effective solution to detect pathogens in blood (21, 27). Furthermore, ddPCR has the potential to dynamically track the progression of these illnesses and provide guidance on the appropriate use of antibiotic drugs (17).

In this study, a duplex droplet digital PCR assay for the detection of BCC and *S. maltophilia* was developed. To our knowledge, there are no reports of rapid, sensitive, and absolute quantification of BCC and *S. maltophilia,* which could cause the BSI outbreak at the same time (6).

## MATERIALS AND METHODS

### Bacterial strains and preparation of DNA templates

The reference strains of BCC (ATCC 25416) and *S. maltophilia* (ATCC 13637) were obtained from the American Type Culture Collection and served as positive control strains in this study. In order to evaluate the specificity of the ddPCR assay, a total of 35 reference and clinical strains (Table S1) were included. Except for BCC and *S. maltophilia*, which were incubated at 30°C, the bacterial strains were cultivated on Brain Heart Infusion (BHI) (Beijing Land Bridge Technology, Beijing, China) agar or Columbia agar plates supplemented with 5% defibrinated sheep blood (Thermo Fisher Scientific, MA, USA). Incubation conditions involved either ambient air or 5% CO_2_ at 37°C, with a duration of 18 to 24 hours. To extract bacterial genomic DNA, the Wizard Genomic DNA Purification Kit (Promega, WI, USA) was employed following the manufacturer’s instructions. Subsequently, the DNA samples were stored at −80°C until further analysis. The Qubit 4 Fluorometer (Thermo Fisher Scientific) was utilized to quantify the genomic DNA.

### Primers and probes

The primers and probes for the ddPCR assay were designed using Primer Express 3.0 software from Applied Biosystems (Foster City, CA, USA) to target specific genes. The *recA* gene (GenBank Accession no.: U70431) was selected for identifying BCC, while the *Smalto* gene (GenBank Accession no.: CP029773) was chosen for detecting *S. maltophilia* (Table 1). Based on the fundamental principles of primer design and verification through fluorescence quantitative PCR using Rotor-Gene Q (Qiagen, Hilden, Germany) with Premix Ex Taq (Probe qPCR; Takara, Dalian, China), the sets that exhibited simultaneous reactions of both primers and probes were ultimately selected from the fifth sets of primers and probes designed by Primer Express 3.0 software. To ensure the specificity of the primers and probes, the BLASTn algorithm and Primer-BLAST (available at https://blast.ncbi.nlm.nih.gov/Blast.cgi) were employed for evaluation. The primers and probes used in this study were synthesized by Beijing Tianyi Huiyuan Biotechnology Co.

**TABLE 1:** Sequences of primers and probes used in this study

Species	Target genes	Sequence and modification (5′−3′)	Length of PCR product (bp)	Reference
*Burkholderia cepacia*	*recA*	F: CGCGTGAAGGTCGTCAAGA	69	(28)
		R: GCCGTACAGGATGTCGAAGATC		
		P: FAM-AAGGTGTCGCCGCCG-BHQ1		
*Stenotrophomonas maltophilia*	*Smalto*	F: ACCTGACGCTGCCGAAAC	57	(29)
		R: TGCGCTTGGCGATGTAAG		
		P: HEX-CGAGCAGGACTACGAC-BHQ1		

### ddPCR assay

The ddPCR experiments were conducted using a QX200 ddPCR system (Bio-Rad, CA, USA). Each ddPCR reaction consisted of a 20 µL reaction mixture, which included 10 µL of 2 × ddPCR SuperMix for Probes (Bio-Rad), 350 nM of probes, 900 nM of forward and reverse primers, 1 µL of template DNA, and nuclease-free water to reach a final volume of 20 µL. Once the reaction mixture was prepared, droplets were generated using a DG8 cartridge and the droplet generator (Bio-Rad). These droplets were then transferred to a 96-well plate and sealed using a PX1 PCR plate sealer (Bio-Rad). The thermal cycling protocol involved an initial denaturation step at 95°C for 10 minutes, followed by 45 cycles of denaturation at 94°C for 30 seconds and annealing/extension at different temperatures for 1 minute. A final extension step was carried out at 98°C for 10 minutes. The temperature ramp speed was set at 2°C/second. Subsequently, the data obtained from the ddPCR experiments were analyzed using QuantaSoft Software (Bio-Rad).

### Optimization and comparison of singleplex and duplex ddPCR assays

We optimized three parameters of singleplex ddPCR, which included the annealing temperature ranging from 50.0°C to 56.7°C, primer concentrations ranging from 300 to 1,000 nM, and probe concentrations ranging from 100 to 800 nM. Once the singleplex ddPCR assay was optimized, we developed a duplex ddPCR assay based on the optimized singleplex protocol. To evaluate the performance of the developed assays, we conducted three parallel reactions using positive reference strains of BCC and *S. maltophilia*. The reactions consisted of (i) singleplex ddPCR, (ii) duplex ddPCR with only one template added, and (iii) duplex ddPCR with both templates added.

### ddPCR assay specificity and sensitivity

To assess the specificity of the duplex ddPCR assay, we conducted reactions using different DNA templates from 35 bacterial pathogen species listed in Table S1. The reactions were performed under the same conditions as described previously. For assessing the sensitivity of the ddPCR assay in this study, the common PCR products of *recA* and *Smalto* genes, which were extracted from the gel using QIAquick Gel Extraction Kit (Qiagen), were cloned into the pMD18-T vector (Takara) and sequenced by Beijing Tianyi Huiyuan Biotechnology Co. with M13 Primers. The plasmids containing *recA* and *Smalto* genes were extracted using Plasmid Mini Kit (Omega, GA, USA) and quantified by Qubit 4 Fluorometer. The copy number of plasmids was calculated as described previously (30). Next, the plasmids of BCC and *S. maltophilia* were serially diluted and six densities from 10^5^ copies/μL to 10° copies/μL were used to determine the sensitivity. Three dilution series were prepared, and dilutions of the same concentrations were mixed to minimize errors during the dilution process.

### The application of ddPCR assay in simulative samples and clinical isolates

BCC and *S. maltophilia* reference strains were incubated in BHI broth overnight at 30°C. The strain solutions were then transferred to 5 mL BHI broth and incubated until reaching an optical density of 0.61 at 600 nm with agitation. The concentrations of BCC and *S. maltophilia* were determined using the plate count method and serial dilution. Artificially contaminated human blood and blood culture bottle samples were mixed with BCC and *S. maltophilia* at seven concentrations ranging from 10^7^ to 10^1^ CFU/mL. For the simulated samples, 100 µL of bacterial solutions with different concentrations were mixed with 900 µL of blood, and then 200 µL of the simulated blood samples were used for DNA extraction without pre-extraction. DNA extraction was performed using two different methods: the Chelex-100 boiling method from Sigma-Aldrich (Sigma, MO, USA) and the kit extraction method using the QIAamp DNA Blood Mini Kit (Qiagen, NRW, Germany). A 1 µL sample of extracted DNA of each bacterial dilution was used as a template to ascertain the limit of detection (LoD). The LoD of the ddPCR assay corresponds to the dilution at which the target DNA can be reliably detected.

For the Chelex-100 boiling method, a 200 µL simulated sample was extracted following the previously described protocol (31), resulting in a final DNA volume of 200 µL. On the other hand, the kit extraction method involved extracting a 200 µL simulated sample according to the kit protocol, resulting in a final DNA volume of 100 µL. Since 1 µL of the template was used, the theoretical bacterial genomic DNA obtained through the kit extraction method is twice as high as that obtained through the Chelex-100 boiling method.

## RESULTS

### Establishment of ddPCR assay and optimization of annealing temperatures

**Fig 1:**
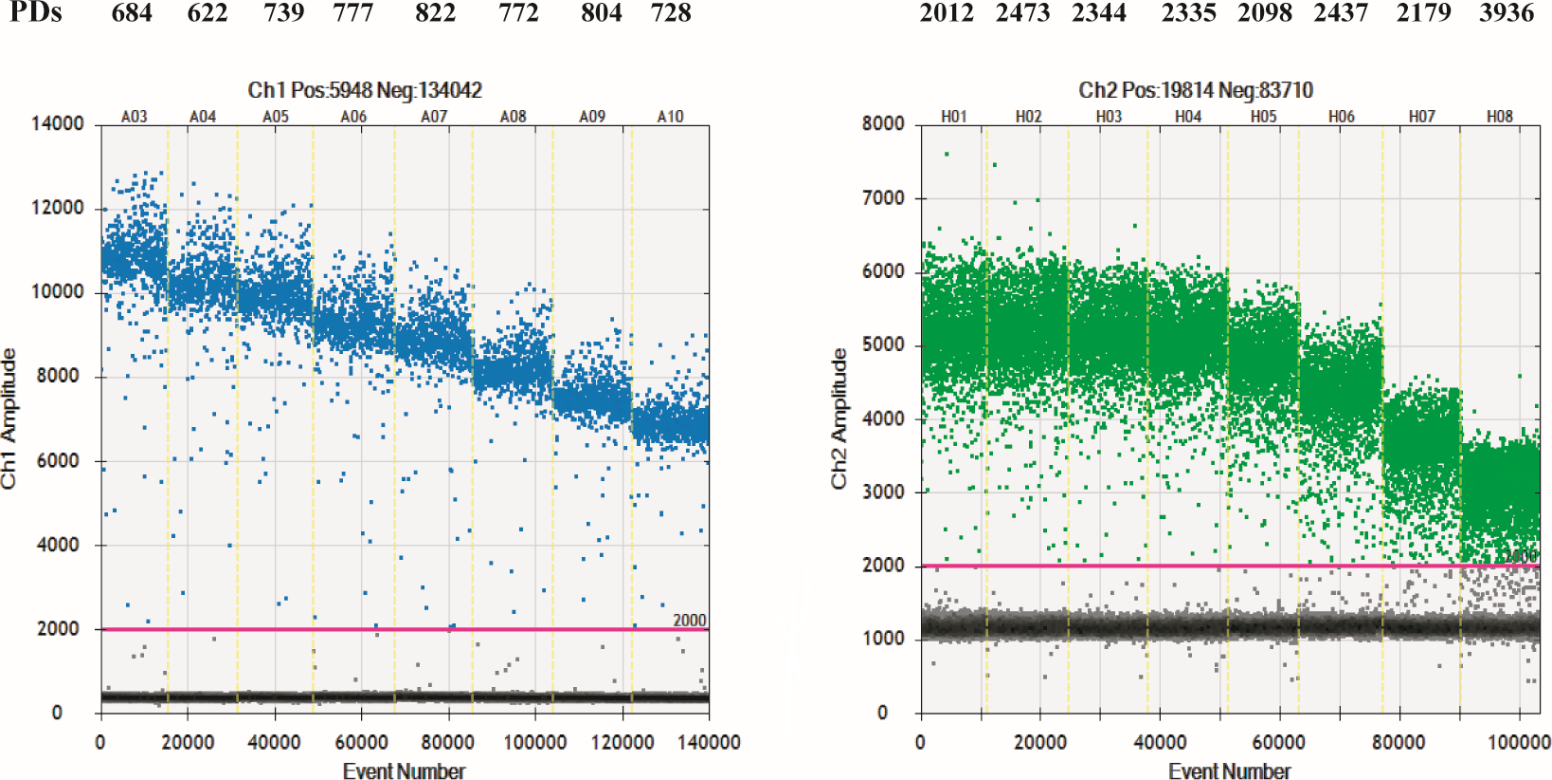
Scatter diagrams illustrating the optimal annealing temperature of the *recA* gene for BCC and the *Smalto* gene for *S. maltophilia* in ddPCR. Ch1, the FAM channel of the *recA* gene; Ch2, the HEX channel of the *Smalto* gene. The horizontal coordinate represents the cumulative number of droplets, and the vertical coordinate represents the fluorescence amplitude. The vertical yellow lines represent the separation of the amplification results. The positive droplets calculated using Poisson statistics based on the number of amplitudes were colored blue and green, respectively. The negative droplets were colored gray. The annealing temperatures of A03–A10 and H01–H08 are 50.0°C, 50.5°C, 51.3°C, 52.2°C, 53.3°C, 54.4°C, 55.6°C, and 56.7°C, respectively. The number of positive droplets at different annealing temperatures was labeled. PDs: positive droplets.

First, the common PCR was conducted using the primers designed in this study at eight different temperatures ranging from 50.0°C to 56.7°C. At all tested temperatures, a single band was consistently observed, indicating successful amplification. The sequencing analysis confirmed the presence of the target genes, including the *recA* gene of BCC and the *Smalto* gene of *S. maltophilia*, which further validated the effectiveness of the primers used in this study. Then, ddPCR was utilized to amplify the *recA* gene and *Smalto* gene at different annealing temperatures (ranging from 50.0°C to 56.7°C), and the results are presented in Fig. 1. For the *recA* gene, positive droplets were obtained at all annealing temperatures. However, at the temperatures of 50.5°C (A04) and 51.3°C (A05), the positive and negative droplets could be distinctly separated into two clusters. Regarding the *Smalto* gene, positive droplets at four annealing temperatures, ranging from 50.0°C to 52.2°C, were significantly distinguished from the negative droplets. The number of amplicons (positive droplets) was recorded as 2,012 at 50.0°C, 2,473 at 50.5°C, 2,344 at 51.3°C, and 2,335 at 52.2°C. To achieve duplex ddPCR assays while ensuring specificity and amplification efficiency, the optimal annealing temperature chosen in this study was 51°C.

### Optimization concentrations of primers and probes of the singleplex ddPCR assay

**Fig 2:**
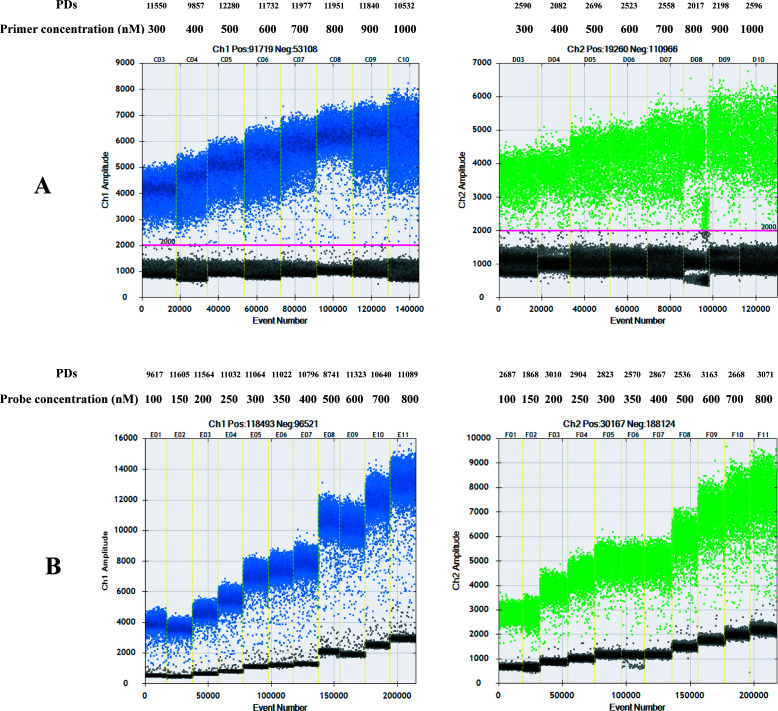
Scatter diagrams of optimal primer and probe concentrations of the singleplex ddPCR assay for detecting BCC and *S. maltophilia*. Ch1, the FAM channel of *recA* gene; Ch2, the HEX channel of the *Smalto* gene. (A) Optimal primer concentrations of the singleplex ddPCR. (B) Optimal probe concentrations of the singleplex ddPCR. The horizontal coordinate represents the cumulative number of droplets, and the vertical coordinate represents the fluorescence amplitude. The vertical yellow lines represent the separation of the amplification results. The positive droplets calculated using Poisson statistics based on the number of amplitudes were colored blue and green, respectively. The negative droplets were colored gray. The number of positive droplets at different annealing temperatures was labeled.

The eight primer concentrations ranging from 300 to 1,000 nM with 100 nM intervals were used to optimize the singleplex ddPCR assay of the *recA* and *Smalto* genes by fixed probe concentration at 300 nM and annealing temperature at 51°C (Fig. 2A). Based on the desired amplification effect and the minimization of scattered positive droplets (“tailing” phenomenon), the primer concentration of 900 nM was used (Fig. 2A). Similarly, for the *recA* gene of BCC and the *Smalto* gene of *S. maltophilia*, a probe concentration of 350 nM was determined as the optimal concentration (Fig. 2B).

### Determination of optimal thermal cycle numbers

To address the issue of the “tailing” phenomenon in the ddPCR assays, we explored the effect of varying the number of thermal cycles. It was observed that increasing the thermal cycle number from 40 to 45 resulted in a more concentrated distribution of positive droplets. This improved concentration facilitates the differentiation between positive and negative droplets (32). Consequently, we have determined that the optimal number of thermal cycles for our study is set at 45 (Fig. S1).

### The optimal reaction system of singleplex and duplex ddPCR

Based on the above results, the optimal duplex ddPCR reaction system determined in this study consists of the following components: 10 µL of 2 × ddPCR SuperMix for Probes (Bio-Rad), 350 nM of probes, 900 nM of forward and reverse primers, 1 µL of template DNA, and nuclease-free water to achieve a final volume of 20 µL. The optimal reaction conditions entail an initial denaturation step at 95°C for 10 minutes, followed by 45 cycles of denaturation at 94°C for 30 seconds and annealing/extension at 51°C for 1 minute, concluding with a final extension step at 98°C for 10 minutes. For the duplex ddPCR assay, the same probe and primer concentrations of the *recA* and the *Smalto* genes were employed.

Based on the results obtained from three parallel reactions with different reference strain templates, it was observed that the duplex ddPCR assay exhibited similar amplification efficiency to that of the singleplex ddPCR (Fig. S2). This indicates that it is indeed feasible to detect two target genes simultaneously in the duplex ddPCR assay.

### Analytical sensitivity and specificity

In order to evaluate the sensitivity of our study, plasmids containing the *recA* gene or *Smalto* gene were diluted into six gradients ranging from 10^5^ to 10° copies/μL. These diluted plasmids, in 1 µL volumes, were used as templates for the duplex ddPCR reactions. The experiments were conducted three times, and the results demonstrated that BCC could be detected as low as 5.35 copies/reaction, while *S. maltophilia* could be detected as low as 7.67 copies/reaction (Table 2).

**TABLE 2:** The sensitivity of the duplex ddPCR reactions^*a*^

Gene name	Expected copies/reaction (cp/r)	The copies of the ddPCR		
		Mean (cp/r)	SD (cp/r)	CV (%)
*recA* (BCC)	5.35 × 10^5^	1.32 × 10^5^	2.65 × 10^3^	2.00
	5.35 × 10^4^	1.16 × 10^4^	9.61 × 10^2^	8.26
	5.35 × 10^3^	2.21 × 10^3^	1.60 × 10^2^	7.27
	5.35 × 10^2^	2.45 × 10^2^	2.53 × 10^1^	10.35
	5.35 × 10^1^	2.33 × 10^1^	4.16 × 10^0^	17.84
	5.35 × 10^0^	9.60 × 10^0^	2.09 × 10^0^	21.75
*Smalto* (*S. maltophilia*)	7.67 × 10^5^	1.22 × 10^5^	2.81 × 10^3^	2.30
	7.67 × 10^4^	2.33 × 10^4^	1.07 × 10^3^	6.90
	7.67 × 10^3^	1.08 × 10^3^	7.18 × 10^1^	6.63
	7.67 × 10^2^	4.63 × 10^2^	2.72 × 10^1^	5.87
	7.67 × 10^1^	4.67 × 10^1^	8.08 × 10^0^	17.32
	7.67 × 10^0^	7.67 × 10^0^	2.00 × 10^0^	21.13

^*a*^ Mean, average copy numbers (*n* = 3) in a final reaction volume of 20 μL. SD, standard deviation; CV, coefficient of variation; CV = SD/mean × 100%.

Furthermore, within the entire effective dynamic range of quantitative detection, the coefficient of variation among the three parallel detection results was found to be less than 25%, indicating good repeatability (Table 2). The standard curve equation for BCC was determined as *y* = 0.2466*x* – 86.345 (*R*² = 0.9998), and for *S. maltophilia*, it was *y* = 0.1575*x* + 2118.6 (*R*² = 0.9915), indicating a strong linear relationship (*R*² > 0.99) in the ddPCR assay for both BCC (in the range of 7.67 × 10^5^–7.67 × 10^0^) and *S. maltophilia* (in the range of 5.35 × 10^5^–5.35 × 10^0^).

To assess the specificity of the duplex ddPCR assay, we employed the genomic DNA of 35 pathogenic bacteria (Table S1) as templates. The results demonstrated that only the genomic DNA of BCC and *S. maltophilia* exhibited positive droplets, whereas the remaining 33 pathogenic bacteria and the negative control displayed only negative droplets. These findings suggest that the ddPCR detection method established in this study exhibits excellent specificity.

### The application of ddPCR assay in simulated and clinical samples

Simulated samples with healthy human blood and blood culture bottles were applied, and two kinds of DNA-extracting methods, including the Chelex-100 boiling method and kit extraction method using the QIAamp DNA Blood Mini Kit, were used and compared. The results indicate that the LoD of BCC in blood culture bottles was 4.00 cp/r (Chelex-100) and 4.87 cp/r (kit extraction), while in healthy human blood, it was 6.13 cp/r (Chelex-100) and 5.00 cp/r (kit extraction) (Table 3). For *S. maltophilia*, the LoD in blood culture bottles was 5.80 cp/r (Chelex-100) and 4.87 cp/r (kit extraction), whereas in healthy human blood, it was 6.93 cp/r (Chelex-100) and 4.40 cp/r (kit extraction) (Table 3).

**TABLE 3:** The results of the duplex ddPCR reactions in simulated blood culture bottle and human blood samples^*a*^

Simulated samples	DNA-extracting methods	Expected CFU/reaction	One target in the reaction			Both targets in the reaction		
			Mean (cp/r)	SD (cp/r)	CV (%)	Mean (cp/r)	SD (cp/r)	CV (%)
Blood culture bottle	*Burkholderia cepacia* ATCC 25416 (Chelex-100）	4.15 × 10^4^	1.79 × 10^4^	3.21 × 10^2^	1.79	1.22 × 10^4^	3.29 × 10^2^	2.69
		4.15 × 10^3^	3.47 × 10^3^	1.63 × 10^2^	4.69	3.15 × 10^3^	2.14 × 10^2^	6.78
		4.15 × 10^2^	3.67 × 10^2^	2.10 × 10^1^	5.72	3.24 × 10^2^	2.71 × 10^1^	8.35
		4.15 × 10^1^	4.13 × 10^1^	5.03 × 10^0^	12.18	3.47 × 10^1^	4.16 × 10^0^	12.01
		4.15 × 10^0^	4.00 × 10^0^	9.20 × 10^−1^	22.91	4.67 × 10^0^	1.01 × 10^0^	21.57
	*Burkholderia cepacia* ATCC 25416 (Qiagen）	9.30 × 10^4^	1.35 × 10^4^	2.31 × 10^2^	1.71	1.35 × 10^4^	5.03 × 10^2^	3.72
		9.30 × 10^3^	1.87 × 10^3^	1.03 × 10^2^	5.50	1.20 × 10^3^	8.00 × 10^1^	6.45
		9.30 × 10^2^	2.39 × 10^2^	2.32 × 10^1^	9.69	2.41 × 10^2^	2.01 × 10^1^	8.34
		9.30 × 10^1^	5.33 × 10^1^	6.11 × 10^0^	11.46	4.87 × 10^1^	8.08 × 10^0^	16.61
		9.30 × 10^0^	4.87 × 10^0^	1.15 × 10^0^	23.73	4.27 × 10^0^	1.01 × 10^0^	23.59
	*S. maltophilia* ATCC 13637 (Chelex-100）	1.63 × 10^4^	3.25 × 10^4^	9.23 × 10^2^	2.84	4.54 × 10^4^	1.36 × 10^3^	2.99
		1.63 × 10^3^	5.27 × 10^3^	1.17 × 10^2^	2.22	4.62 × 10^3^	1.44 × 10^2^	3.12
		1.63 × 10^2^	5.31 × 10^2^	3.19 × 10^1^	6.01	5.30 × 10^2^	3.27 × 10^1^	6.18
		1.63 × 10^1^	6.07 × 10^1^	7.57 × 10^0^	12.48	6.13 × 10^1^	9.87 × 10^0^	16.09
		1.63 × 10^0^	5.80 × 10^0^	1.40 × 10^0^	24.14	5.73 × 10^0^	1.29 × 10^0^	22.43
	*S. maltophilia* ATCC 13637 (Qiagen）	3.26 × 10^4^	2.57 × 10^4^	8.80 × 10^2^	3.43	2.77 × 10^4^	7.86 × 10^2^	2.84
		3.26 × 10^3^	4.70 × 10^3^	2.39 × 10^2^	5.06	4.75 × 10^3^	2.14 × 10^2^	4.51
		3.26 × 10^2^	4.39 × 10^2^	3.59 × 10^1^	8.17	4.88 × 10^2^	3.74 × 10^1^	7.66
		3.26 × 10^1^	3.27 × 10^1^	6.11 × 10^0^	18.70	3.00 × 10^1^	4.00 × 10^0^	13.33
		3.26 × 10^0^	4.87 × 10^0^	1.21 × 10^0^	24.77	6.53 × 10^0^	1.55 × 10^0^	23.78
Healthy human blood	*Burkholderia cepacia* ATCC 25416 (Chelex-100）	4.15 × 10^4^	1.12 × 10^4^	1.50 × 10^2^	1.06	1.12 × 10^4^	3.13 × 10^2^	2.80
		4.15 × 10^3^	1.52 × 10^3^	4.61 × 10^1^	3.04	1.54 × 10^3^	5.29 × 10^1^	3.44
		4.15 × 10^2^	1.36 × 10^2^	9.17 × 10^0^	6.74	2.07 × 10^2^	1.55 × 10^1^	7.52
		4.15 × 10^1^	2.33 × 10^1^	3.06 × 10^0^	13.09	6.73 × 10^1^	7.57 × 10^0^	11.25
		4.15 × 10^0^	6.13 × 10^0^	1.51 × 10^0^	24.69	5.73 × 10^0^	1.29 × 10^0^	22.43
	*Burkholderia cepacia* ATCC 25416 (Qiagen）	9.30 × 10^4^	1.28 × 10^4^	4.00 × 10^2^	3.13	1.27 × 10^4^	5.03 × 10^2^	3.97
		9.30 × 10^3^	1.56 × 10^3^	1.31 × 10^2^	8.41	3.38 × 10^3^	1.82 × 10^2^	5.39
		9.30 × 10^2^	1.67 × 10^2^	1.96 × 10^1^	11.73	1.81 × 10^2^	1.65 × 10^1^	9.13
		9.30 × 10^1^	3.80 × 10^1^	7.21 × 10^0^	18.98	2.73 × 10^1^	3.06 × 10^0^	11.18
		9.30 × 10^0^	5.00 × 10^0^	1.22 × 10^0^	24.33	5.70 × 10^0^	1.37 × 10^0^	24.12
	*S. maltophilia* ATCC 13637 (Chelex-100）	1.63 × 10^4^	2.11 × 10^4^	4.16 × 10^2^	1.98	2.49 × 10^4^	4.16 × 10^2^	1.67
		1.63 × 10^3^	2.29 × 10^3^	6.43 × 10^1^	2.81	2.46 × 10^3^	8.00 × 10^1^	3.25
		1.63 × 10^2^	2.09 × 10^2^	1.33 × 10^1^	6.38	2.29 × 10^2^	1.97 × 10^1^	8.60
		1.63 × 10^1^	7.67 × 10^1^	8.08 × 10^0^	10.54	5.73 × 10^1^	9.24 × 10^0^	16.11
		1.63 × 10^0^	6.93 × 10^0^	1.51 × 10^0^	21.84	3.93 × 10^0^	9.50 × 10^−1^	24.03
	*S. maltophilia* ATCC 13637 (Qiagen）	3.26 × 10^4^	1.57 × 10^4^	3.25 × 10^2^	2.07	1.60 × 10^4^	3.33 × 10^2^	2.08
		3.26 × 10^3^	1.31 × 10^3^	5.03 × 10^1^	3.83	1.55 × 10^3^	8.33 × 10^1^	5.38
		3.26 × 10^2^	1.41 × 10^2^	1.22 × 10^1^	8.65	1.48 × 10^2^	1.20 × 10^1^	8.11
		3.26 × 10^1^	1.80 × 10^1^	2.00 × 10^0^	11.11	3.13 × 10^1^	4.16 × 10^0^	13.29
		3.26 × 10^0^	4.40 × 10^0^	1.06 × 10^0^	24.05	6.60 × 10^0^	1.64 × 10^0^	24.80

^*a*^ Mean, average copy numbers (*n* = 3) in a final reaction volume of 20 μL. SD, standard deviation; CV, coefficient of variation; CV = SD/mean × 100%; Chelex-100, Chelex-100 boiling method; Qiagen, QIAamp DNA Blood Mini Kit.

In simulated blood culture bottle samples, the standard curve equations for BCC were *y* = 0.4234*x* + 451.78 (*R*² = 0.9913, Chelex-100) and *y* = 0.1611*x* + 161.19 (*R*² = 0.9987, kit extraction). For *S. maltophilia*, the standard curve equations were *y* = 1.97*x* + 537.95 (*R*² = 0.9962, Chelex-100) and *y* = 0.7762*x* + 551.91 (*R*² = 0.9931, kit extraction). In simulated human blood samples, the standard curve equation was *y* = 0.2681*x* + 105.04 (*R*² = 0.9988, Chelex-100) and *y* = 0.1534*x* + 83.822 (*R*² = 0.9996, kit extraction) for BCC and *y* = 1.2917*x* + 57.859 (*R*² = 0.9999, Chelex-100) and *y* = 0.483*x* − 64.084 (*R*² = 0.9997, kit extraction) for *S. maltophilia*. Therefore, a strong linear relationship (*R*² > 0.99) was observed between BCC and *S. maltophilia* in simulated samples.

A total of 58 clinical samples from blood culture bottles were tested, comprising eight samples that flagged positive results and 50 samples that did not by Blood Culture Systems. Among the eight samples that tested positive, one sample was positive for BCC, while another sample was positive for *S. maltophilia*. These two samples were subsequently isolated into respective strains.

## DISCUSSION

BCC and *S. maltophilia* are significant nosocomial pathogens associated with BSI and antimicrobial resistance (4–6). Compared to the culture method, molecular biology methods, particularly the ddPCR method, have optimized the detection process, allowing for the timely and accurate identification of pathogens in BSIs (17, 20). In this study, we have developed a duplex ddPCR assay that enables simple and accurate detection of BCC and *S. maltophilia* in BSIs. Our literature review revealed a lack of existing duplex ddPCR assays specifically designed for identifying BCC and *S. maltophilia* in BSIs. To simplify experimental conditions and enable simultaneous detection of both genes, we optimized the annealing temperature and determined 51°C as the optimal reaction temperature. We also established the optimal concentrations of primers and probes for the singleplex ddPCR assay, as well as the optimal thermal cycle numbers. The optimal reaction conditions were 45 cycles, 350 nM of probes, and 900 nM of forward and reverse primers for both BCC and *S. maltophilia*. Based on those optimal reaction conditions, the optimal duplex ddPCR reaction system was determined and the feasibility of the parallel reactions was verified using the reference strains of BCC and *S. maltophilia*.

This duplex ddPCR assay for BCC and *S. maltophilia* had high sensitivity and could detect as low as 5.35 copies/reaction of BCC and 7.67 copies/reaction of *S. maltophilia* with good repeatability. The specificity of the duplex ddPCR assay was demonstrated for 33 other bacterial pathogens, and no ampliﬁcation was detected for those bacterial pathogens. Thus, the specificity of this duplex ddPCR assay was 100%. The sensitivity of the duplex ddPCR assay for BCC and *S. maltophilia* was at the same order of magnitude in artificially contaminated human blood and blood culture bottle samples, which is similar to that of plasmid standards. Simulated samples designed for blood culture bottles explored the possibility of using the ddPCR method for the detection of BCC and *S. maltophilia* during the blood culture process, which can improve detection sensitivity while saving samples. Additionally, we compared two nucleic acid extraction methods, the Chelex-100 boiling method and the kit extraction method, and the results showed similar detection sensitivity for both methods. The Chelex-100 boiling method has the advantages of simplicity, short processing time, and low cost compared to the kit extraction method. Furthermore, this method leverages the advantage of the low template quality requirement of ddPCR, suggesting its potential application in BSIs. The duplex ddPCR method established in this study was used to detect 58 clinical blood culture samples, and the results were consistent with the culture results, further demonstrating the potential application of this method in clinical samples. A recent study revealed that the LoDs of ddPCR were 7.6 and 1.4 copies for the *B. cepacia* PC783 and *Burkholderia cenocepacia* J2315 strains, respectively, while those of qPCR were 25.9 and 28.2 Ct, equivalent to 76.4 and 14.4 copies, respectively (25). For both strains, ddPCR exhibited 10 times higher sensitivity than qPCR. Moreover, the ddPCR results of this study’s experiment were consistent with our investigations. mNGS has emerged as a powerful tool for identifying the causative pathogens of BSIs. However, ddPCR has a higher positivity rate than mNGS and blood cultures (83.3%, 68.3%, and 16.7%, respectively) (20). A clinical research study indicated that patients who tested positive by ddPCR alone had milder clinical symptoms and better clinical outcomes than those who tested positive by blood culture and ddPCR (33). Therefore, in the early stages of BSIs, ddPCR may be a quick and accurate way to determine the causative microorganisms and direct therapy choices.

This study has several limitations that should be acknowledged. First, the clinical application of the ddPCR assay was only tested on a relatively small sample size of 58 blood bottle samples. Therefore, to enhance the reliability of the results, it is necessary to further validate them by expanding the sample size, particularly with the inclusion of clinical blood samples. Second, since SM and BCC are more commonly found in lower respiratory tract samples, it would be beneficial for future research to explore their detection in these types of specimens.

## Data Availability

The data that support the findings of this study are openly available in Genbank with the reference number CP007787 for the *recA* gene of *Burkholderia cepacia* and CP029773 for *Smalto* gene of *Stenotrophomonas maltophilia*.

## SUPPLEMENTAL MATERIAL

Supplemental material spectrum.03569-23-s0001.pdf Table S1; Fig. S1 and S2.

ASM does not own the copyrights to Supplemental Material that may be linked to, or accessed through, an article. The authors have granted ASM a non-exclusive, world-wide license to publish the Supplemental Material files. Please contact the corresponding author directly for reuse.
