# Supplementary material for: Development of a duplex droplet digital PCR assay for the detection of Burkholderia cepacia complex and Stenotrophomonas maltophilia in bloodstream infections
Source: Microbiol Spectr. 2024 Feb 27;12(4):e03569-23. doi: 10.1128/spectrum.03569-23 (PMC10986617; doi:10.1128/spectrum.03569-23)
Supplement: Supplemental material — Table S1; Fig. S1 and S2. [file spectrum.03569-23-s0001.pdf]

Supplemental Table. Strains used in this study

Bacteria	Strain no./source[a]
Stenotrophomonas maltophilia	ATCC 13637
Burkholderia cepacia	ATCC 25416
Enteroinvasive *E. coli*	CMCC 44825
Enterotoxigenic *E. coli*	10407
Uropathogenic *E. coli*	UPEC 536
Citrobacter freundii	ATCC 8090
Pseudomonas aeruginosa	ATCC 27853
Salmonella paratyphi A	CMCC 50001
S. typhimurium	CMCC 50013
S. enterica subsp. enterica	ATCC BAA-664
Klebsiella pneumoniae subsp. Pneumoniae	ATCC 700603
K. pneumoniae	CMCC 46117
Vibrio parahemolyticus	ATCC 17802
Enterococcus faecium	CGMCC 1.2135
E. faecalis	CGMCC 1.2136
E. durans	CGMCC 1.2284
E. avium	CGMCC 1.2505
E. hirae	CGMCC 1.2140
E. mundtii	CGMCC 1.2486
Acinetobacter baumannii	ATCC 17978

Staphylococcus aureus subsp. aureus	ATCC 43300
Streptococcus alactolyticus	ATCC 43077
S. pyogenes	ATCC 19615
S. salivarius	L.1001111
Listeria monocytogenes	ATCC BAA-679
Yersinia enterocolitica	Pa134
Streptococcus gallolyticus	ATCC 43143
S. pasteurianus	ATCC 43144
S. pneumoniae	ATCC 49619
S. suis	SC84
Shigella dysenteriae	CMCC 51329
Sh. flexneri	301
Sh. bogdii	CMCC 51105
Sh. sonnei	CMCC 51081
Enterobacter cloacae	ATCC 700323

[a] ATCC, American Type Culture Collection, CGMCC, China General Microbiological Culture Collection Center, CMCC, National Center for Medical Culture Collections.

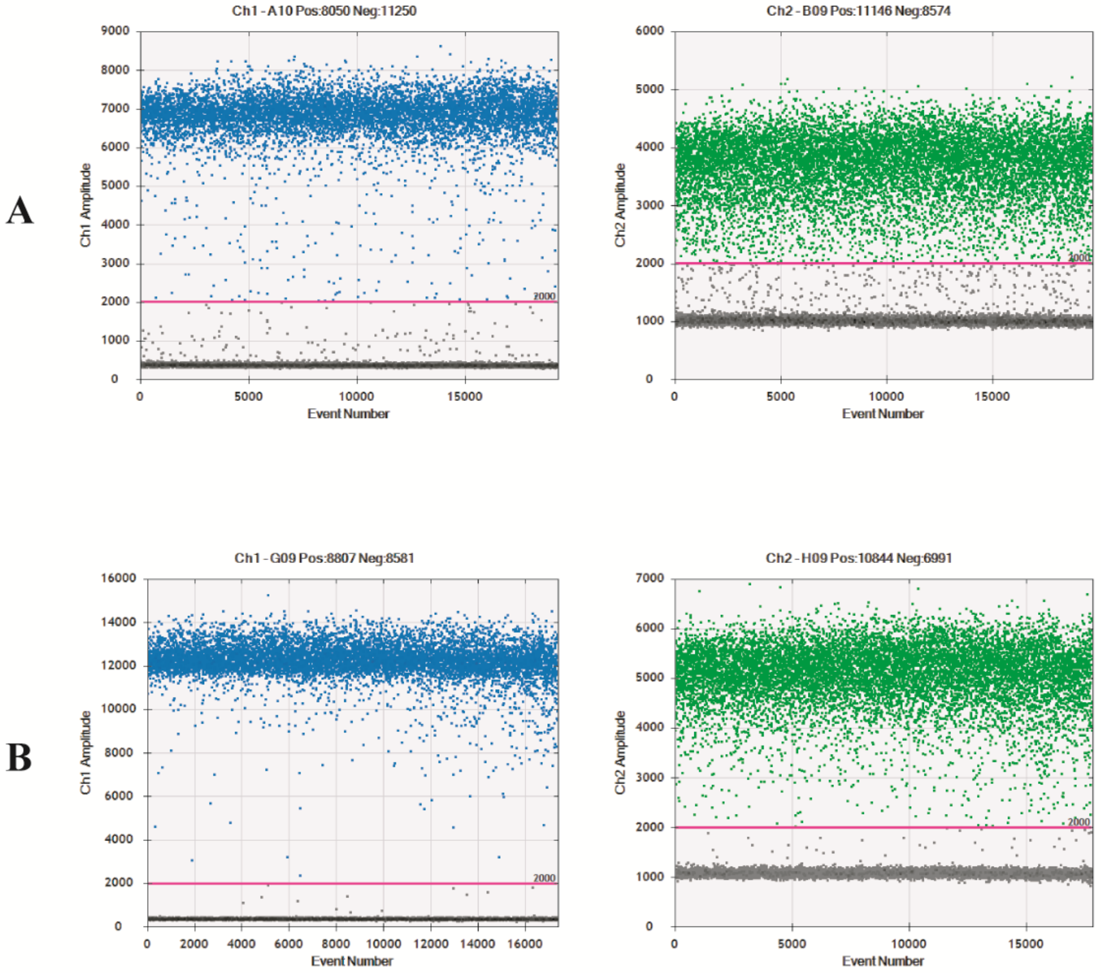

Supplemental Figure 1. Scatter diagrams of optimal thermal cycle numbers of the singleplex ddPCR assay for detection *Burkholderia cepacia* Complex and *Stenotrophomonas maltophilia*

Ch1, the FAM channel for BCC; Ch2, the HEX channel for *S. maltophilia.* A, the thermal cycle numbers were 40; B, the thermal cycle numbers were 45. The horizontal coordinate represents the cumulative number of droplets, and the vertical coordinate represents the fluorescence amplitude. The positive droplets calculated using Poisson statistics based on the number of amplitudes were colored blue and green, respectively. The negative droplets were colored grey.

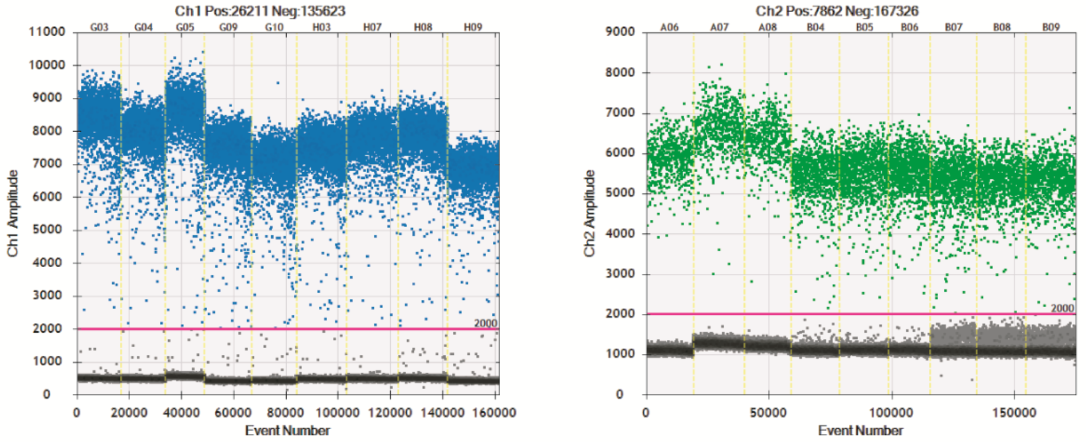

Supplemental Figure 2. Scatter diagrams were generated to compare the parallel reactions using both duplex and singleplex ddPCR assays with reference strains of BCC and *S. maltophilia*.

Ch1, the FAM channel for BCC; Ch2, the HEX channel for *S. maltophilia.* The reference strains were simultaneously detected in three parallel reactions: (i) the singleplex ddPCR (G01–G05, A06–A08); (ii) the duplex ddPCR with only one template (G094–H03, B04–B06); and (iii) the duplex ddPCR with both templates (H07–H09, B07–B09). The horizontal coordinate represents the cumulative number of droplets, and the vertical coordinate represents the fluorescence amplitude. The vertical yellow lines represent the separation of the amplification results. The positive droplets calculated using Poisson statistics based on the number of amplitudes were colored blue and green, respectively. The negative droplets were colored grey.
